# Oxidative stress and antioxidant parameters in neutropenic patients secondary to chemotherapy

**DOI:** 10.12669/pjms.322.9200

**Published:** 2016

**Authors:** Akif Dogantekin, Ali Gurel, Bilal Ustundag, Selcuk Ilhan, Emin Tamer Elkiran

**Affiliations:** 1 Dr. Akif Dogantekin, MD. Department of Internal Medicine, Emek Hospital, Gaziantep, Turkey; 2 Dr. Ali Gurel, MD. Mengucek Gazi Training and Research Hospital, Department of Nephrology, Erzincan, Turkey; 3 Prof. Bilal Ustundag, MD. Department of Biochemistry, Firat University Medical School, Elazig, Turkey; 4 Selcuk Ilhan, MD. Associate Professor, Department of Pharmacology, Firat University Medical School, Elazig, Turkey; 5 Prof. Emin Tamer Elkiran, MD. Department of Medical Oncology, Inonu University Medical School, Malatya, Turkey

**Keywords:** Antioxidant, Malondialdehyde, Neutropenia secondary to chemotherapy, Oxidative stress, Paraoxonase

## Abstract

**Objective:**

Neutropenia is a serious adverse event that necessitates dosage reduction in patients receiving chemotherapy. In this study, we evaluated the oxidative stress and antioxidant parameters in neutropenic patients after chemotherapy both during the neutropenic period and after successful treatment of neutropenia with filgrastim.

**Methods:**

We studied paraoxonase (PON1), arylesterase (ARE), malondialdehyde (MDA), high-density lipoprotein (HDL), lactate dehydrogenase (LDH), and alkaline phosphatase (ALP) in addition to routine biochemical and hematologic parameters. SPSS 12.0 was used for statistical evaluation of data (SPSS, Chicago, IL, USA).

**Results:**

In our study, PON1, HDL, and LDH levels during the period of active neutropenia were statistically significantly higher than these levels were after resolution of neutropenia (P<0.05); MDA and ALP levels were statistically significantly lower during the period of active neutropenia (P<0.05).

**Conclusions:**

Overall, free oxygen radicals (FOR) were increased and antioxidant parameters were decreased with resolution of neutropenia. This is probably due to FOR produced by the increased number of neutrophils rather than tumor burden.

## INTRODUCTION

Cancer is a disease that usually originates in a specific organ system and is characterized by uncontrolled proliferation of malignant cells at a faster rate than that of normal cells, deterioration of differentiation, infiltration into surrounding tissues, and metastasis to different parts of the body.^1^ Oxidative stress has been shown to increase the clinical progression of cancer. Malondialdehyde (MDA) is the primary product of peroxidation of polyunsaturated fatty acid and also the most studied. MDA is mutagenic, genotoxic, and carcinogenic due to these features. Endogenous and exogenous antioxidants may inhibit cancer growth by neutralizing or blocking the effects of free oxygen radicals (FOR).^2^

Paraoxonase (PON1) is a lipophilic antioxidant, connected to an HDL cholesterol, weight of 43 kDa and found in serum and the liver. The antioxidant role of PON1 is associated with the protective effect of low-density lipoprotein (LDL) cholesterol from oxidation. Two common functional polymorphisms of PON1 were determined and this polymorphism affects the serum PON1 activity. Although PON1 and arylesterase (ARE) have been characterized as two separate enzymes, studies have shown that in human serum one gene product has both PON1and ARE enzyme activity. ARE is a thiol enzyme that is reactivated by 2-mercaptoethanol and cysteine and acts on phenyl acetate to release phenol.^3^

Chemotherapy provides healing and induces significant remission in a variety of cancer types. However, cancer chemotherapy regimens have adverse effects on the hematopoietic system. Neutropenia is a disorder in which a patient has a low absolute neutrophil count in comparison to a healthy individual. An absolute neutrophil count (ANC)<500/mm^3^ (neutropenia), and especially of<100/mm^3^ (severe neutropenia), leads to a tendency to develop serious infections. Treatment with myelotoxic chemotherapy frequently leads to neutropenia.^4^

Neutropenia is the primary cause of dose reductions due to adverse events during chemotherapy, and can also lead to postponing treatment, decreasing chemotherapy’s positive outcomes. Febrile neutropenia is a life-threatening oncologic emergency situation that often requires use of antibiotics and hospitalization and reduces quality of life.^4^ Filgrastim (recombinant human granulocyte colony stimulating factor (G-CSF)) is used to reduce the incidence of neutropenia associated with bone marrow–suppressing chemotherapy in patients with cancer.^5^

In this study, we aimed to evaluate the oxidant (MDA) and antioxidant (PON1 and ARE) parameters in patients with neutropenia secondary to chemotherapy in the neutropenic period (ANC< 1000/mm^3^) and after the elimination of neutropenia with use of filgrastim (leukocyte count> 10,000/mm^3^), in order to understand the oxidative stress status in these conditions. According to our literature search, this is perhaps the first study evaluating the relationship between neutropenia/GCS-F and MDA, PON1 and ARE activities.

## METHODS

We obtained ethic approval from the local Ethics Committee before starting the study; written informed consent was obtained from all patients. This study included patients with neutropenia secondary to chemotherapy. To enroll, patients had to be greater than 18 years of age, have developed neutropenia after chemotherapy, and have no dietary practices or history of smoking or drug use that could have affected serum analysis. Name, age, sex, presence of fever, and histopathologic diagnosis of patients were recorded. Venous blood samples were taken from the antecubital vein during the period of neutropenia and after the elimination of neutropenia with filgrastim after at least 8-12 hours of fasting. Biochemistry tubes and ethylenediaminetetracetic acid (EDTA) tubes were used for collecting blood samples. Blood samples were centrifuged for five minutes at 4000 rpm, and serum and plasma were separated. Samples were stored in the freezer at -20ºC until analysis.

MDA analysis was performed with a spectrophotometric method modified from Satoh and Yagi. PON1 activity was measured by spectrophotometric evaluation of 4-nitrophenol, which is the enyzmatic product of the substrate paraoxon (O, O-diethyl-Op-nitrophenyl phosphate; Sigma Co., London, UK). ARE activity was measured by spectrophotometric evaluation of phenol, which is the enyzmatic product of the substrate phenyl acetate (Sigma). LDH and ALP enzymatic activities were measured by using commercial kits in an Olympus AU2700™ Chemistry-Immuno Analyzer (Olympus America; Melville, NY, USA) using a spectrophotometric method. Serum HDL levels were measured using commercial kits in the autoanalyzer.

The data obtained in this study were expressed as mean± standard error. SPSS 12.0 statistical package software was used for statistical evaluation of data (SPSS Inc). The difference between means was evaluated using a paired Student’s *t*-test. *P* values <0.05 were considered statistically significant.

## RESULTS

A total of 48 patients were enrolled in the study, 25 male (52%) patients and 23 females (48%). Patients’ mean age was 56.12± 11.77. 29 of the 48 patients (60.4%) had metastases; 20 of 48 (41.7%) had fever.

**Fig.1:**
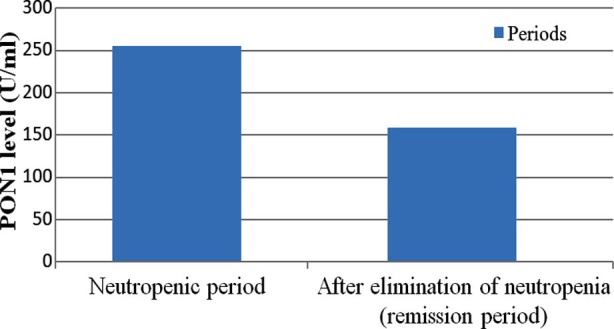
Changes of PON1 levels between periods.

MDA levels during the neutropenic period were statistically significantly lower than during remission (*P*= 0.008), as were ALP levels (*P*= 0.020). Conversely, PON1 and ARE levels during the neutropenic period were statistically significantly higher than during remission (*P*= 0.017 and *P*= 0.003, respectively), as were HDL levels (*P*= 0) and LDH levels (*P*= 0.029). (Table-I and Fig.1).

**Table-I:** MDA, PON1, ARE, HDL, LDH, ALP levels.

	Neutropenic period	After elimination of neutropenia (remission period)	P value
MDA	0.82 ± 0.03	0.92 ± 0.03	0.008 (n= 48)
PON1	256.11 ± 44.43	158.88 ± 35.44	0.017(n= 48)
ARE	140.31 ± 33.44	53.20 ± 10.51	0.003 (n= 43)
HDL	30.15 ± 1.90	20.50 ± 1.64	0 (n= 48)
LDH	280.12 ± 25.90	229.04 ± 14.58	0.029 (n= 48)
ALP	81.88 ± 8.59	107.81 ± 7.47	0.020 (n= 48)

## DISCUSSION

Systemic chemotherapy plays an important role in the treatment of cancer patients when a given cancer is incurable using regional treatment methods. Combination chemotherapies provide improvement and significant remission in most cases for patients with cancer. Adverse effects on the hematopoietic system are common in patients receiving chemotherapy regimens. This bone marrow suppression may cause anemia and neutropenia, reducing both patients’ quality of life and efficacy of treatment. Hospital care and treatment costs for anemia and neutropenia are substantial. Neutropenia may require reductions of chemotherapy dose or even postponement of treatment. Patients with neutropenia are likely to develop febrile neutropenia, which is usually a life-threatening condition, reduces quality of life, and requires hospitalization and intravenous antibiotics. Therefore, prevention of the bone-marrow suppressive effects of cancer therapy is important to enhance quality of life and increase the effectiveness of treatment.^4^

Filgrastim (recombinant G-CSF) has long been used for reduction of neutropenia associated with bone-marrow suppressive chemotherapy in cancer patients. Current data indicate that G-CSF not only increases the formation of the granulocyte colony from bone marrow precursor cells, but also enhances the functioning of neutrophils via superoxide anion production.^6^

Noriyuki et al.^7^ found that G-CSF increased myeloperoxidase activity of neutrophils in non-insulin dependent patients with diabetes mellitus and improved impaired production of free oxygen radicals (FOR) in neutrophils in diabetic patients with poor glycemic control. This study revealed that G-CSF may be used in patients with poorly controlled diabetes with impaired neutrophil function in order to decrease bacterial infections, which increase morbidity and mortality. Additionally, due to the positive effects on neutrophils G-CSF is being used to prevent infections in acquired immunodeficiency syndrome, myelodysplastic syndrome, and congenital agranulocytosis.^8^

Although G-CSF is a hematopoietic glycoprotein, current studies have revealed that it has some other interesting properties. G-CSF is known to be cardioprotective and, discovered to prevent mitochondrial injury caused by oxidative stress in cardiac tissue.^9^

Kojima et al.^10^ revealed that, in mouse oxygen-induced retinopathy model, with its anti-oxidant properties, G-CSF reduced vascular obliteration, neovascularization and, protected neural retina from oxidative damage.

Free radicals are short life-span, unstable, low-molecular-weight, highly active atoms or molecules with several unpaired electrons. FOR are produced continuously during normal cell metabolism and neutralized by the antioxidant defense system. However, oxidative stress occurs with production of excessive amounts of FOR or a decrease in antioxidant defense systems. Oxidative stress plays a critical role in carcinogenesis and tumor growth via DNA damage, chromosomal aberration, mutations in tumor suppressor genes, uncontrolled cell division, and genomic instability.^11^

Over the past 20 years, MDA has been accepted as a marker of lipid peroxidation and MDA levels have been measured and evaluated in various diseases. The main source of MDA in biological samples is the peroxidation of polyunsaturated fatty acids. In stress conditions, MDA is derived from prostaglandin-like endoperoxides. In this case, MDA is a biomarker of lipid peroxidation, and also a potential cause of cancer. Patients with breast or lung cancer have shown to have higher plasma MDA levels.^12^ MDA formation in breast cancer has been demonstrated in another study in which the mean value of MDA increased; intra-day variability was also found to be more powerful. MDA levels are also higher in patients with cervical cancer compared to healthy individuals.^13^ Plasma MDA levels were determined to increase in stomach cancer in association with the disease stage.^14^ Higher plasma MDA levels in patients with chronic lymphocytic leukemia were observed in several studies.^15^ MDA–protein interactions were detected in malignant melanoma and non-melanoma skin cancer.^16^

PON1 is an esterase considered to be synthesized and secreted in the liver in humans and has the ability to hydrolyze paraoxon. Human serum PON1 is physically associated with HDL. Purified PON1 is a glycosylated protein of approximately 43 kD molecular weight.^17^,^18^ The physiological role of PON1 is thought to be the hydrolysis of lipid peroxides and the protection of LDL against oxidative cellular damage caused by toxic agents.^19^ PON1 is an enzyme that also has an important role in the antioxidant system in liver microsomes.^20^

PON1 also has ARE activity, but ARE activity remains independent of changes in PON1 activity. PON1 activity increases and ARE activity decline with increasing concentrations of sodium chloride. In many diseases, serum PON1 levels change along with increases in oxidative stress.^21^ Akcay et al.^22^ studied the relationship between plasma lipoproteins and PON1 in pancreatic and gastric cancer patients in two different studies. In patients with pancreatic cancer, HDL and PON1 levels were lower than in healthy subjects. Results in patients with gastric cancer were similar to those found with pancreatic cancer.^22^,^23^ Elkiran et al.^11^ found decreased serum PON1 levels and PON1/HDL ratios in patients with lung cancer.

In the study of Nishikawa et al.^24^ in pediatric patients with acute lymphoblastic leukemia, FOR were found to be high at the beginning of the disease before decreasing to normal levels with induction chemotherapy. FOR formed by the breakdown of malignant cells may be cleared with adequate hydration by the kidneys.

Neutrophils constitute major cellular defense against invading microorganisms. After contact with opsonized microorganism, phagocytosis occurs. The destroyer ability of neutrophils relies on the antimicrobial proteins and peptides and on generation of FOR. FOR production is keystone for the progression of many inflammatory diseases. Neutrophils are the main source of FOR and thus oxidation and many cellular signaling mechanisms promotes.^25^,^26^

In our study, MDA levels during the neutropenic period were statistically significantly lower than during remission (*P*=0.008). PON1, ARE, and HDL levels of neutropenic period were statistically significantly higher than those during remission (*P*=0.017, *P*=0.003, and *P*=0, respectively).

## CONCLUSION

Oxidative damage caused by FOR plays an important role in the pathogenesis of many malignant diseases. FOR are vital factors in the stimulation of cancer formation. Organisms try to protect themselves from oxidative damage through antioxidants. In this study, MDA levels increased and antioxidant enzymes PON1 and ARE levels (along with HDL levels) decreased after the resolution of neutropenia secondary to chemotherapy. Endogenous sources of FOR include mitochondria, cytochrome P450 metabolism, peroxisomes, inflammatory cell activation, neutrophils, eosinophils and macrophages;exogenous sources include environmental factors such as non-genotoxic carcinogens.^27^ In this study FOR increased after successful treatment of neutropenia with G- CSF/filgrastim. Although it is mentioned in the literature that G- CSF administration reduces oxidative stress in disorders which are associated with oxidative stress; this may probably be attributed to FOR produced by the increased number of neutrophils induced by filgrastim rather than tumor burden. The limitations of our study are relatively small sample size and lack of information about the last status of the patients. Different studies on this issue may be helpful to fully understand and support our findings.
